# Avoidant/Restrictive Food Intake and Selective Eating in Children: Clinical Profile, Nutritional Deficiencies, and Behavioral Correlates in a Tertiary Pediatric Center

**DOI:** 10.3390/nu18132059

**Published:** 2026-06-24

**Authors:** Livia Gargiullo, Valentina Colistra, Annalisa Grandin, Rosaria Marotta, Italo Pretelli, Ludovica Ricci, Mariangela Irrera, Antonio Musolino, Isabella Tarissi de Jacobis, Maria Rosaria Marchili, Alberto Villani

**Affiliations:** 1 General Pediatrics, Infectious Diseases and Level II Pediatric Emergency Department Unit, Bambino Gesù Children’s Hospital, IRCCS, 00152 Rome, Italy; 2 Anorexia and Eating Disorders Unit, Child and Adolescent Psychiatry Unit, Bambino Gesù Children’s Hospital, IRCCS, 00152 Rome, Italy; 3 Pediatrics Residency Program, Department of Pediatrics, University of Rome Tor Vergata, 00100 Rome, Italy

**Keywords:** ARFID, selective eating, picky eating, food selectivity, pediatric nutrition, micronutrient deficiency, transdiagnostic, BMI, ultra-processed foods, mealtime behavior

## Abstract

**Background**: Avoidant/restrictive food intake disorder (ARFID) and selective eating are increasingly recognized in pediatric nutrition, but food selectivity has been predominantly studied in dedicated eating disorder settings and in underweight children, potentially underestimating its prevalence across broader clinical populations. This study aimed to characterize food selectivity as a transdiagnostic feature in children referred to a tertiary pediatric nutrition center, regardless of referral diagnosis or BMI status. **Methods**: This retrospective observational study included 417 consecutive children and adolescents (median age 9.3 years, IQR 4.1–12.9; 47.5% male) assessed at the General Pediatric Eating Disorders Outpatient Unit of Bambino Gesù Children’s Hospital IRCCS, Rome, Italy, between May 2024 and April 2026. Food selectivity was defined as clinician-documented avoidance of at least one of four food groups (vegetables, fruit, fish, and legumes). Patients were classified as having primary selective eating/ARFID (Group A, n = 141), unrecognized selective eating (Group B, n = 163), or no selectivity (Group C, n = 113). **Results**: Food selectivity was identified in 293 patients (70.3%), including 70.8% of those referred for obesity or overweight and 50.0% of those referred for eating disorders. Prevalence did not differ across BMI categories (*p* = 0.554), confirming that selective eating is independent of anthropometric status. Ferritin deficiency showed a significant gradient across groups (Group A 32.2%, Group B 17.9%, Group C 10.8%; *p* = 0.002). Screen use during meals and ultra-processed food consumption were similarly elevated in Groups A and B and significantly higher than in Group C (*p* = 0.002 and *p* < 0.001, respectively), with no difference between the two selective groups. **Conclusions**: Food selectivity is a transdiagnostic and BMI-independent feature affecting the majority of children referred for pediatric nutritional evaluation. Children with unrecognized selective eating share the same nutritional risks and behavioral correlates as those formally diagnosed with ARFID, supporting the integration of a brief food group avoidance screen into routine nutritional assessment regardless of the primary referral diagnosis.

## 1. Introduction

Avoidant/Restrictive Food Intake Disorder (ARFID), introduced in the DSM-5 in 2013, encompasses persistent eating disturbances leading to inadequate nutritional and/or energy intake, resulting in weight loss or growth faltering, nutritional deficiencies, dependence on supplements or enteral feeding, and/or significant psychosocial impairment [1]. Unlike anorexia nervosa, food restriction in ARFID is not driven by body image concerns, but rather by limited interest in eating, fear of aversive consequences, or sensory-based avoidance of foods [1,2]. Among these presentations, sensory-related selective eating—commonly referred to as picky or selective eating—has emerged as one of the most prevalent and clinically relevant manifestations in pediatric populations [1,3].

Selective eating is characterized by the rejection of specific foods or entire food groups based on sensory characteristics such as texture, color, smell, or taste, often resulting in markedly restricted dietary variety [3,4]. Although mild picky eating is considered relatively common during early childhood and frequently transient, severe or persistent food selectivity may lead to clinically significant nutritional, developmental, and psychosocial consequences [5]. Recent population-based data suggest that avoidant/restrictive food intake phenotypes are substantially more common than previously recognized, with persistent restrictive eating behaviors affecting a considerable proportion of children across development [6]. Importantly, food selectivity exists along a continuum ranging from normative developmental behaviors to severe restrictive eating patterns consistent with ARFID [5,7]. The way food selectivity is operationalized differs widely across studies, ranging from single-item parent-reported picky eating to structured screening questionnaires such as the Nine-Item ARFID Screen (NIAS) and to clinician-led dietary assessment of avoidance toward individual food groups [1,3]. This methodological heterogeneity is a major source of the wide variability in reported prevalence estimates and clinical correlates and limits direct comparison across cohorts, underscoring the need to clearly specify the assessment method adopted in each study.

Several studies have demonstrated that selective eating may occur independently of low body weight or overt malnutrition [3,8]. Children with highly restricted diets can present with normal or even elevated BMI despite significant micronutrient deficiencies and poor dietary diversity [8,9]. Severe food selectivity has been associated with deficiencies in vitamins A, B12, C, D, E, iron, zinc, folate, and calcium, as well as severe complications such as scurvy, rickets, and impaired psychosocial functioning [2,8,10]. Critically, anthropometric parameters alone are insufficient to capture the nutritional risk associated with selective eating: BMI-based assessment may systematically underdetect micronutrient vulnerability in this population, as growth parameters do not adequately reflect diet quality or micronutrient adequacy [8,10]. This concept is particularly relevant in pediatric clinical practice, where selective eating behaviors are often underestimated in children without underweight or growth impairment.

Food selectivity is especially prevalent among neurodevelopmental populations, particularly children with autism spectrum disorder (ASD), in whom sensory sensitivities and rigid eating patterns frequently contribute to restrictive dietary intake [11,12]. However, selective eating is not limited to ASD or to patients formally diagnosed with ARFID. Emerging evidence suggests that restrictive eating behaviors may be highly prevalent across a broad range of pediatric feeding and eating presentations, including children referred for obesity, growth faltering, gastrointestinal symptoms, and other nutritional concerns [6,11]. In tertiary pediatric nutrition centers, where patients are referred across a wide spectrum of primary diagnoses, the true prevalence of co-occurring food selectivity likely remains substantially underestimated, as systematic screening for selective eating is rarely performed outside of dedicated eating disorder settings.

Previous literature on ARFID has largely focused on tertiary eating disorder settings, where patients often present with severe malnutrition or low BMI [3]. Consequently, selective eating has traditionally been conceptualized primarily as a distinct diagnostic subgroup rather than as a transdiagnostic behavioral feature. However, recent evidence increasingly supports the idea that selective eating behaviors may represent a clinically meaningful dimension that cuts across diagnostic categories and nutritional phenotypes [5,6]. In particular, the presence of food selectivity toward even a single food group may contribute to reduced dietary diversity, impaired nutritional adequacy, and maladaptive mealtime behaviors, regardless of the primary diagnosis or anthropometric status. Behavioral and environmental factors—including screen exposure during meals, consumption of ultra-processed foods, and parental feeding strategies—may further influence the persistence and severity of food refusal behaviors, contributing to maladaptive feeding dynamics across diagnostic presentations [7,13].

The present study aimed to characterize the clinical profile, nutritional deficiencies, and behavioral correlates of children referred to a tertiary pediatric nutrition center, exploring food selectivity as a cross-cutting feature defined by the presence of avoidance toward at least one food group, regardless of referral diagnosis or BMI status. We hypothesized that selective eating would be highly prevalent across all diagnostic categories and nutritional phenotypes, supporting its recognition as a transdiagnostic clinical dimension warranting systematic assessment in all children referred for nutritional evaluation.

## 2. Materials and Methods

### 2.1. Study Design and Setting

This was a retrospective observational study conducted at the General Pediatric Eating Disorders Outpatient Unit of Bambino Gesù Children’s Hospital IRCCS, Rome, Italy, a tertiary-level pediatric referral center. Data were collected from consecutive nutritional assessments performed across outpatient and day hospital settings between May 2024 and April 2026. The study was conducted in accordance with the Declaration of Helsinki. Informed consent for the use of clinical data for research purposes was obtained as part of the general privacy and data processing consent signed by patients’ caregivers upon admission to Bambino Gesù Children’s Hospital IRCCS, in accordance with Italian privacy law (Legislative Decree 196/2003 and EU Regulation 2016/679).

### 2.2. Participants

All children and adolescents who underwent a nutritional assessment during the study period were included. No additional inclusion or exclusion criteria were applied, in order to capture the full spectrum of presentations referred for nutritional evaluation in a real-world tertiary setting.

### 2.3. Data Collection

Clinical data were collected prospectively as part of routine nutritional assessments by the multidisciplinary team of the General Pediatric Eating Disorders Outpatient Unit. At each visit, the following information was recorded using a structured clinical form:

- Demographic and anthropometric data: date of birth, date of assessment, sex, body weight (kg), standing height (m), and body mass index (BMI, kg/m^2^).
- Referral diagnosis: the primary reason for referral as documented by the referring clinician, subsequently categorized for analysis into the following groups: Selective eating/ARFID, Growth faltering, Eating disorders, Obesity, Overweight, and Other.
- Perinatal history: type of conception (spontaneous or medically assisted), course of pregnancy, gestational age (weeks), birth weight (kg).
- Early feeding history: breastfeeding (yes/no), exclusive breastfeeding for six months (yes/no), type of complementary feeding introduction (classical/baby-led weaning/mixed), and regularity of the weaning process.
- Gastrointestinal history: bowel habit (regular/constipated/diarrheic/alternating), gastro-esophageal reflux (yes/no), and food allergies/intolerances (yes/no).
- Current dietary habits: number of daily meals (≥5 vs. <5), breakfast consumption, lunch outside the home, at least one daily family meal, meal preparation with children, consumption of sugar-sweetened beverages, and consumption of ultra-processed foods. All dietary-habit variables were recorded as binary items (yes/no) by the clinician during a structured interview with the caregiver, capturing the presence or absence of each habitual practice rather than a quantified intake frequency. Breakfast consumption, lunch regularly eaten outside the home, and routine meal preparation together with the child were each coded as habitual (yes) or not (no); habitual consumption of sugar-sweetened beverages and of ultra-processed foods (the latter defined according to the NOVA classification) was likewise coded as present or absent. For these two items, “consumption” denoted the regular presence of the food category in the child’s habitual diet (daily or near-daily intake reported by the caregiver) rather than occasional intake. Daily physical activity was defined as caregiver-reported habitual active play or movement on most days of the week, and organized sport/motor activity as regular participation in a structured sport or motor programme; both were recorded as yes/no. For the analysis, these two items were combined into a composite indicator of any physical activity, defined as participation in daily active play and/or organized sport (assessable in children with data on at least one of the two items).
- Food selectivity: presence of selectivity toward each of four food groups (vegetables, fruit, fish, and legumes), recorded as yes/no by the clinician based on dietary history obtained from the caregiver and, where applicable, the child.
- Behavioral and lifestyle variables: co-sleeping (yes/no and duration), sleep–wake rhythm regularity, daily screen time (≤2 h/>2 h per day), screen use during meals (yes/no), daily physical activity (yes/no), and organized sport or motor activity (yes/no).
- Nutritional biomarkers: results of laboratory tests performed as part of routine clinical care, including ferritin, 25-hydroxyvitamin D (25(OH)D), vitamin C, and folate, recorded as binary variables (deficiency present: yes/no) according to standard age-appropriate laboratory reference ranges.

### 2.4. Anthropometric Assessment and BMI z-Score

BMI was calculated as weight divided by height squared (kg/m^2^). BMI z-scores were calculated for children aged 2–20 years using the CDC 2000 Growth Charts reference data, applying the Box–Cox power transformation (LMS method) as implemented in the validated age- and sex-specific reference charts developed by Shypailo (Shypailo RJ, 2020. Age-based Pediatric Growth Reference Charts. Baylor College of Medicine, Children’s Nutrition Research Center, Body Composition Laboratory. Available at: http://www.bcm.edu/bodycomplab/BMIapp/BMI-calculator-kids.html (accessed on 23 April 2026)). Weight status was classified as underweight (<5th percentile), normal weight (5th–84th percentile), overweight (85th–94th percentile), or obese (≥95th percentile), according to the CDC 2000 age- and sex-specific reference charts.

### 2.5. Definition of Food Selectivity

For the purposes of this study, food selectivity was defined as the presence of clinician-documented avoidance of at least one of the four assessed food groups (vegetables, fruit, fish, and legumes), regardless of the primary referral diagnosis or BMI status. This operational definition was applied to the entire cohort in order to identify selective eating as a cross-cutting behavioral feature, independent of whether selective eating was the primary reason for referral. The ≥1-food-group threshold was adopted as an inclusive screening criterion, prioritizing sensitivity for any clinically relevant food avoidance rather than the confirmation of a formal diagnosis. To assess the robustness of this operational choice, the main findings (prevalence, independence from BMI, and behavioral correlates) were re-examined under stricter thresholds (avoidance of ≥2, ≥3, and all four food groups).

Based on this definition, participants were classified into three groups:

- Group A—Primary selective eating: patients referred with a primary diagnosis of selective eating, ARFID, food refusal, or restrictive eating (n = 141);
- Group B—Unrecognized selective eating: patients referred for a diagnosis other than selective eating, but presenting with documented selectivity toward at least one food group (n = 163). The primary referral diagnoses of Group B were growth faltering, obesity, overweight, eating disorders, and other conditions;
- Group C—Non-selective: patients referred for a diagnosis other than selective eating, with no selectivity documented for any of the four food groups (n = 113).

### 2.6. Statistical Analysis

Categorical variables are presented as absolute frequencies and percentages. Continuous variables are presented as mean ± standard deviation (SD) or median and interquartile range (IQR), depending on the distribution. Normality was assessed using the Shapiro–Wilk test. Comparisons between groups for categorical variables were performed using the chi-square test or Fisher’s exact test, as appropriate. Continuous variables were compared using the Kruskal–Wallis test for three-group comparisons, with post hoc pairwise Mann–Whitney U tests where applicable. The association between food selectivity (present/absent, regardless of diagnosis) and BMI category was assessed using the chi-square test. Statistical significance was set at *p* < 0.05 (two-tailed). All analyses were performed using Python (version 3.12; Python Software Foundation) with the pandas, numpy, and scipy libraries. Effect sizes were reported for the principal comparisons (Cramér’s V for categorical associations and the rank-biserial correlation for continuous comparisons), each with 95% confidence intervals. The association between food selectivity and the main behavioral correlates was additionally examined using multivariable logistic regression adjusted for age and sex, with results expressed as adjusted odds ratios and 95% confidence intervals. As a sensitivity analysis, the prevalence of food selectivity and its associations with BMI category and behavioral variables were re-estimated under stricter operational thresholds (avoidance of ≥2, ≥3, and all four food groups).

## 3. Results

### 3.1. Study Population and Referral Diagnoses

A total of 417 children and adolescents were included in the study (47.5% male, 52.5% female), with a median age of 9.3 years (IQR 4.1–12.9; range 0.4–18.0 years). Patients were referred across three clinical settings: outpatient clinic (n = 157, 37.6%), day hospital (n = 220, 52.8%), and neuropsychiatric consultation (n = 38, 9.1%), and two outpatient records with incomplete setting data (n = 2). The distribution of primary referral diagnoses and the prevalence of food selectivity within each diagnostic category are shown in Table 1. The most frequent referral diagnoses were selective eating/ARFID (n = 141, 33.8%), growth faltering (n = 88, 21.1%), and obesity/overweight (n = 89, 21.3%).

### 3.2. Prevalence of Food Selectivity Across the Entire Cohort

**Figure 1:**
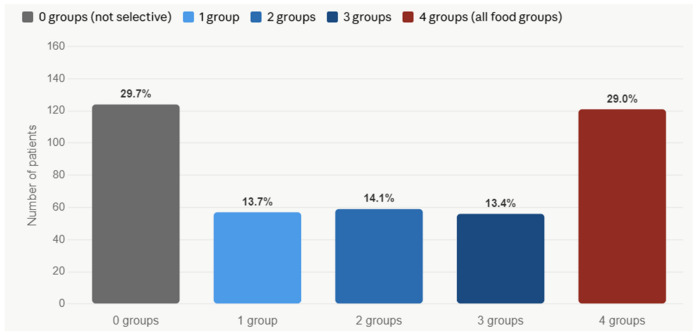
Distribution of number of food groups avoided in the entire cohort (N = 417).

Food selectivity, defined as clinician-documented avoidance of at least one of four assessed food groups (vegetables, fruit, fish, and legumes), was identified in 293 of 417 patients (70.3%), irrespective of the primary referral diagnosis. The distribution by number of food groups avoided is shown in Figure 1. Notably, 29.0% of the entire cohort avoided all four food groups simultaneously, while 29.7% showed no documented selectivity.

**Figure 2:**
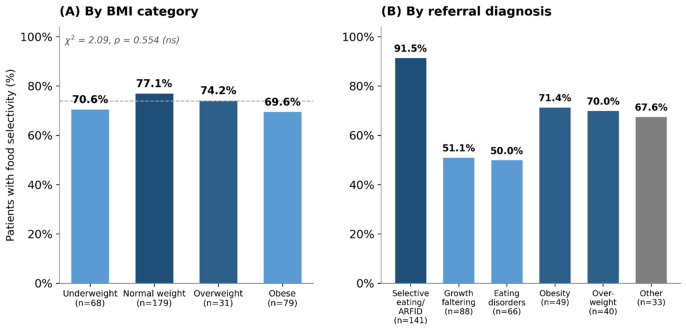
Prevalence of food selectivity (≥1 food group): (**A**) across BMI categories in children aged ≥2 years (N = 357), and (**B**) across referral diagnoses. In panel (**A**), prevalence did not differ across BMI categories (χ^2^ = 2.09, *p* = 0.554).

Selectivity was highly prevalent across all diagnostic categories (Table 1). Among children referred for obesity or overweight, 70.8% presented with documented food selectivity for at least one group; among those referred for eating disorders, 50.0% showed selectivity; and among those referred for growth faltering, 51.1% were selective for at least one food group. Crucially, the prevalence of food selectivity did not differ significantly across BMI categories (underweight 70.6%, normal weight 77.1%, overweight 74.2%, obese 69.6%; χ^2^ = 2.09, *p* = 0.554), confirming that food selectivity is independent of anthropometric status (Figure 2). In sensitivity analyses applying stricter thresholds, food selectivity was present in 56.5% of the cohort when defined as avoidance of ≥2 food groups, 42.3% when defined as ≥3 food groups, and 29.0% when defined as avoidance of all four groups.

### 3.3. Group Characteristics

Based on the operational definition described in Section 2.5, patients were classified into three groups:

- Group A—Primary selective eating (n = 141, 33.8%): 56.7% male, mean age 7.1 ± 3.9 years; BMI z-score –0.72 ± 1.34.
- Group B—Unrecognized selective eating (n = 163, 39.1%): 44.8% male, mean age 9.6 ± 4.7 years; BMI z-score +0.35 ± 1.86.
- Group C—Non-selective (n = 113, 27.1%): 40.4% male, mean age 9.6 ± 6.0 years; BMI z-score −0.03 ± 1.90.

Demographic and anthropometric characteristics of the three groups are summarized in Table 2.

Age differed significantly across the three groups (Kruskal–Wallis H = 23.9, *p* < 0.001). BMI z-score differed significantly (Kruskal–Wallis H = 14.2, *p* < 0.001), driven by the higher BMI z-score in Group B compared to Group A (Mann–Whitney *p* < 0.001), reflecting the inclusion of obese and overweight patients in Group B. No significant difference in BMI z-score was found between Group B and Group C (*p* = 0.318). Group A showed a significantly lower BMI z-score than Group C (Mann–Whitney *p* = 0.005; rank-biserial r = 0.23).

### 3.4. Pattern of Food Selectivity by Group

The mean number of food groups avoided was 3.1 ± 1.3 in Group A, 2.4 ± 1.2 in Group B, and 0 by definition in Group C. In Group A, 55.3% of patients avoided all four food groups simultaneously, compared to 26.4% in Group B (*p* < 0.001).

The prevalence of selectivity for each individual food group is shown in Table 3. Vegetables were the most universally rejected category across both selective groups (Group A 84.4%, Group B 77.9%), with no statistically significant difference between groups (*p* = 0.198). In contrast, selectivity for fruit, fish, and legumes was significantly more prevalent in Group A than in Group B (fruit: 69.5% vs. 50.9%, *p* = 0.001; fish: 77.3% vs. 59.5%, *p* = 0.001; legumes: 74.5% vs. 54.6%, *p* < 0.001).

### 3.5. Nutritional Deficiencies

Nutritional biomarkers were available for a subset of patients, with variable rates of missing data across groups. Among patients with available data, deficiency rates differed across the three groups, reaching statistical significance only for ferritin (Table 4).

Ferritin deficiency showed a statistically significant gradient across groups (Group A 32.2% [28/87], Group B 17.9% [22/123], Group C 10.8% [9/83]; *p* = 0.002), with a clear stepwise decrease from primary to non-selective patients. Vitamin D deficiency was highly prevalent across all groups (Group A 33.8%, Group B 36.5%, Group C 26.9%) with no significant difference (*p* = 0.374). Vitamin C deficiency followed a non-significant gradient (Group A 31.8%, Group B 24.3%, Group C 21.4%; *p* = 0.391). Folate deficiency was documented in more than three quarters of patients tested in both Group A (74.3%, 26/35) and Group B (75.9%, 22/29), compared to 59.1% in Group C (*p* = 0.363). With the exception of ferritin, the remaining comparisons did not reach statistical significance, likely due to the high proportion of missing biomarker data.

### 3.6. Perinatal and Early Feeding History

Perinatal characteristics did not differ significantly between groups for most variables. Complicated pregnancy was reported in 20.3% of Group A, 17.9% of Group B, and 15.8% of Group C (*p* = 0.621). Preterm birth (<37 weeks) was documented in 12.5% of patients with available data, with no significant difference across groups.

Irregular weaning was significantly more frequent in Group A (32.3%) than in Group B (16.9%; *p* = 0.002). Breastfeeding rates were similar across groups (Group A 70.9%, Group B 68.1%, Group C 65.8%; *p* = 0.623). Gastro-esophageal reflux was reported in 11.3% of Group A, 9.2% of Group B, and 8.8% of Group C (*p* = 0.721). Constipation was present in 38.3% of Group A, 31.5% of Group B, and 26.3% of Group C (*p* = 0.141).

### 3.7. Behavioral and Lifestyle Correlates

Behavioral variables are reported in Table 5. Screen use during meals was markedly prevalent in both Group A (58.4%) and Group B (57.1%), compared to Group C (33.8%; *p* = 0.002 for overall comparison). Similarly, consumption of ultra-processed foods was highly prevalent in both selective groups (Group A 62.3%, Group B 63.6%) compared to non-selective patients (Group C 41.4%; *p* = 0.001), with Groups A and B showing near-identical rates.

In age- and sex-adjusted logistic regression models (Table 6), food selectivity (avoidance of ≥1 food group) was independently associated with screen use during meals (adjusted OR 2.50, 95% CI 1.47–4.26; *p* = 0.001), ultra-processed food consumption (aOR 2.87, 95% CI 1.79–4.58; *p* < 0.001), sugar-sweetened beverage consumption (aOR 2.32, 95% CI 1.25–4.29; *p* = 0.007) and co-sleeping (aOR 2.02, 95% CI 1.14–3.58; *p* = 0.016). Effect sizes for the three-group comparisons were small-to-moderate (Cramér’s V: screen use during meals 0.21, 95% CI 0.10–0.33; ultra-processed foods 0.19, 0.10–0.30; sugar-sweetened beverages 0.14, 0.06–0.24; co-sleeping 0.22, 0.13–0.34).

Consumption of sugar-sweetened beverages was more commonly reported in selective patients (Group A 30.8%, Group B 25.5%) than in Group C (15.5%; *p* = 0.031). Co-sleeping showed a significant gradient across groups (Group A 60.4%, Group B 47.4%, Group C 31.3%; *p* < 0.001). Preparation of meals together with the child was paradoxically more common in Group A (27.6%) than in Group B (14.8%; *p* = 0.043). No significant differences were found between groups for the composite physical activity measure, number of daily meals, or breakfast consumption.

## 4. Discussion

The present study examined food selectivity as a cross-cutting behavioral feature in a large consecutive cohort of children and adolescents referred to a tertiary pediatric nutrition center, irrespective of their primary referral diagnosis. The interpretation below is organized around three issues raised by the data: the high prevalence and transdiagnostic nature of food selectivity, its independence from BMI at the screening (≥1-group) threshold, and the shared behavioral profile of recognized and unrecognized selective eaters. Together, these findings support the reconceptualization of food selectivity as a transdiagnostic clinical dimension that warrants systematic assessment across all referral contexts in pediatric nutrition practice.

### 4.1. Prevalence and Transdiagnostic Nature of Food Selectivity

The 70.3% prevalence of food selectivity observed in our cohort substantially exceeds rates reported in community-based populations, where persistent picky eating affects an estimated 15–20% of children [6]. This discrepancy likely reflects both the tertiary referral context—where more severe and complex presentations cluster—and the broad operational definition adopted, which captured selectivity toward any of four fundamental food groups. Crucially, the bimodal distribution of the number of food groups avoided—with peaks at zero and four groups—suggests that food selectivity in clinically referred children tends to be either absent or pervasive, with relatively few patients avoiding only one or two groups in isolation. This all-or-nothing pattern has not been previously described in tertiary pediatric nutrition settings and may reflect the severity of sensory-based avoidance in children who require specialist care.

The finding that 70.8% of children referred for obesity or overweight presented with documented food selectivity is particularly noteworthy. This observation challenges the traditional clinical framing, in which selective eating and excess weight are implicitly treated as mutually exclusive nutritional presentations. Emerging evidence suggests that highly selective diets—despite their caloric adequacy or excess—may paradoxically contribute to obesity through the preferential consumption of energy-dense, nutritionally poor foods, including ultra-processed products and sugar-sweetened beverages [8]. Our data support this hypothesis: obese and overweight children with selectivity showed the same elevated prevalence of ultra-processed food consumption and sugar-sweetened beverage intake as children with primary selective eating. This convergence suggests that selective eating in the context of excess weight may represent a distinct phenotype characterized by qualitative dietary restriction rather than quantitative excess, and deserves dedicated clinical attention.

The 50% prevalence of selectivity among children referred for eating disorders is consistent with recent evidence highlighting the clinical overlap between ARFID and other eating disorder presentations [10]. It also raises the possibility that food selectivity may contribute to the maintenance of restrictive eating behaviors in some eating disorder patients, independent of body image concerns, and should be assessed routinely in this population.

It might be argued that avoidance of a single food group reflects factors other than clinically relevant selectivity, such as family dietary habits, food availability, allergy or intolerance, or individual taste preferences. We consider these explanations unlikely to account for the avoidance patterns observed in our cohort. The four food groups assessed (vegetables, fruit, fish and legumes) were selected precisely because they are major sources of essential nutrients, so that the complete avoidance of any one of them is unlikely to be physiological. Food allergies, which were systematically investigated in this cohort, are an improbable cause of the complete exclusion of an entire food group. Likewise, while restricted family dietary habits and taste-based preferences may contribute to selective eating, neither justifies the elimination of a whole, nutrient-relevant food group: a reduced acceptance of individual foods on a sensory basis can be developmentally normative, whereas the avoidance of an entire food group is not. Finally, although food selectivity has been reported to aggregate within families, such familial patterning does not render it physiological. The sensitivity analyses applying stricter thresholds further support the robustness of this definition: whereas selectivity for ≥1 group was independent of BMI, more pervasive selectivity (≥2, ≥3, or all four groups) became progressively less common at higher BMI, indicating that the inclusive threshold captures a clinically meaningful behavioral pattern rather than incidental single-food avoidance.

### 4.2. Food Selectivity Is Independent of BMI

One of the most clinically important findings of this study is the absence of any significant association between food selectivity and BMI category (*p* = 0.554). Across all weight categories—from underweight to obese—approximately 70–77% of children showed selectivity for at least one food group. This finding is consistent with previous reports demonstrating that children with highly restricted diets can present with normal or elevated BMI despite significant micronutrient deficiencies [3,8,9]. It directly challenges the implicit clinical heuristic that BMI screening is sufficient to identify children at nutritional risk due to selective eating.

The implication for clinical practice is substantial: if food selectivity were screened only in children with low BMI or growth faltering, approximately 70% of selective eaters in a tertiary nutrition setting would remain unidentified. This estimate is corroborated by our Group B data: 163 patients (39.1% of the entire cohort) had documented selectivity for at least one food group but were referred for a different primary diagnosis and would not typically have triggered a formal ARFID evaluation. The concept of unrecognized selective eating—food avoidance that is clinically present but not the primary referral concern—represents a meaningful gap in current clinical assessment practice and has, to our knowledge, not been formally characterized in the previous literature.

### 4.3. Vegetable Refusal as a Universal Feature

Across both selective groups, vegetables were the most consistently rejected food category (Group A 84.4%, Group B 77.9%), with no significant difference between groups (*p* = 0.198). This universality of vegetable refusal—present at comparable rates regardless of whether selectivity was the primary diagnosis—suggests that vegetable avoidance may represent the most accessible screening marker for food selectivity in pediatric clinical encounters. In contrast, avoidance of fruit, fish, and legumes was significantly more prevalent in Group A, indicating a dose–response relationship between diagnostic severity and the number of food groups affected.

### 4.4. Ferritin Deficiency as a Gradient of Selectivity Severity

Ferritin deficiency showed a statistically significant gradient across the three groups (Group A 32.2%, Group B 17.9%, Group C 10.8%; *p* = 0.002), confirming that iron stores are progressively more compromised with increasing food selectivity severity. This finding is consistent with the established association between restricted dietary variety and iron deficiency in pediatric populations [2,8]. Notably, Group B—children with unrecognized selectivity—showed an intermediate ferritin deficiency rate (17.9%), significantly higher than Group C, highlighting that even selectivity that does not constitute the primary clinical concern carries measurable nutritional consequences.

Vitamin D deficiency was highly prevalent across all three groups (27–37%), with no significant between-group difference. This pattern is consistent with the widespread prevalence of vitamin D insufficiency in Italian pediatric populations regardless of dietary pattern [2], and likely reflects both latitude-related reduced sun exposure and the generally low dietary vitamin D content in children’s diets in this age range.

The high rate of missing biomarker data—particularly for folate and vitamin C—is an important limitation of this study and reflects the real-world variability in laboratory testing in a busy tertiary center, where testing is ordered based on clinical indication rather than a standardized protocol. The true prevalence of nutritional deficiencies in this cohort is therefore likely underestimated, particularly in Group B, where the absence of a primary selective eating diagnosis may have reduced clinical suspicion and laboratory testing rates.

### 4.5. Behavioral Correlates: Screen Use During Meals and Ultra-Processed Foods

The near-identical prevalence of screen use during meals in Group A (58.4%) and Group B (57.1%)—both significantly higher than Group C (33.8%, *p* = 0.002)—is one of the most clinically actionable findings of this study. Screen use during meals has been associated with reduced parental sensitivity to child hunger and satiety cues, impaired interoceptive awareness, and decreased dietary variety in young children [7,13]. Our data suggest that this behavioral correlate is not specific to formally diagnosed ARFID but is equally prevalent in children with unrecognized selectivity, raising the hypothesis that shared environmental mealtime dynamics may contribute to the persistence of food avoidance across diagnostic categories.

Similarly, the high and comparable prevalence of ultra-processed food consumption in Groups A and B (>62% in both) versus Group C (41.0%, *p* < 0.001) is consistent with the hypothesis that selective eaters systematically preferentially accept ultra-processed foods, whose uniform texture, predictable taste, and reduced sensory complexity may lower the sensory threshold for acceptance [8]. The clinical paradox is that access to ultra-processed foods may reduce mealtime conflict in the short term, while reinforcing dietary restriction and displacing micronutrient-rich whole foods in the longer term.

Co-sleeping, present in 60.4% of Group A and 47.4% of Group B compared to 31.3% of Group C (*p* < 0.001), showed a significant gradient that mirrors the severity gradient of food selectivity. While the causal direction of this association cannot be determined from our cross-sectional data, co-sleeping may serve as a proxy for broader parenting styles characterized by high accommodation of child distress, which have been previously associated with the persistence of avoidant feeding behaviors [13]. The paradoxically higher rate of meal preparation with the child in Group A (27.6%) compared to Group B (14.8%) is consistent with this interpretation: caregivers of children with recognized selective eating may have adopted food exposure strategies as a compensatory response, whereas caregivers in Group B—unaware of the selectivity—have not yet modified their feeding practices accordingly.

### 4.6. Irregular Weaning as an Early Correlate

The significantly higher rate of irregular weaning in Group A (32.3%) compared to Group B (16.9%; *p* = 0.002) suggests that early complementary feeding difficulties may represent a developmental precursor of the more pervasive and clinically recognized selective eating phenotype. Although the retrospective nature of weaning data precludes causal inference, this finding is consistent with longitudinal evidence linking early feeding difficulties to persistent selective eating in later childhood [5,7]. Importantly, irregular weaning was not significantly more prevalent in Group B compared to Group C, suggesting that the early feeding trajectory may differentiate children who will develop primary selective eating from those in whom selectivity emerges as a secondary feature associated with other primary diagnoses.

### 4.7. Clinical Implications

The data presented here support a fundamental shift in the clinical approach to selective eating in pediatric nutrition settings. Rather than restricting food selectivity assessment to children with a formal ARFID diagnosis or low BMI, the findings suggest that a brief, structured screening for food group avoidance should be integrated into the routine nutritional assessment of all children referred to pediatric nutrition services, regardless of the primary referral diagnosis. Given the simplicity of the four-item food group selectivity screen used in this study, such integration would be feasible within standard clinical workflows and would allow the identification of the approximately 39% of patients with unrecognized selectivity who currently receive no targeted intervention for their food avoidance.

Furthermore, the behavioral correlates identified—screen use during meals, ultra-processed food consumption, and co-sleeping—represent modifiable targets for multidisciplinary intervention. Their cross-diagnostic prevalence suggests that behavioral counseling addressing mealtime environment and parental feeding strategies should be offered to families of all children with documented food selectivity, independently of the primary clinical diagnosis.

### 4.8. Limitations

Several limitations of this study should be acknowledged. First, the cross-sectional design precludes causal inference regarding the relationship between behavioral variables and food selectivity. Second, food selectivity was assessed using a four-item clinician-documented binary screen, which, while clinically practical, does not capture the full complexity of selectivity severity, sensory sensitivity profiles, or associated psychosocial impairment. Third, micronutrient dosing was not performed uniformly across patients: laboratory testing was ordered at the discretion of the referring clinician and varied according to the type of clinical access (outpatient vs. day hospital) and the specific clinical indication, rather than following a standardized protocol. As a consequence, the proportion of patients with available biomarker data differed substantially across groups and across analytes, limiting the precision of nutritional deficiency estimates and potentially introducing selection bias if testing was preferentially performed in children with more severe or symptomatic presentations. In particular, folate was measured in only a small subset of patients, and the high prevalence of folate deficiency observed in both selective groups—although clinically concerning—must be interpreted with caution given the very limited sample size and the non-random nature of testing. Fourth, the study was conducted in a single tertiary-level center, which may limit generalizability to community or primary care settings, where the clinical profile of selective eating may differ. Fifth, the retrospective nature of perinatal and early feeding data introduces potential recall bias.

Despite these limitations, this study provides, to our knowledge, the first systematic characterization of unrecognized food selectivity as a transdiagnostic feature in a large consecutive pediatric nutrition cohort, and offers clinically actionable evidence supporting universal selectivity screening in tertiary pediatric nutrition practice.

## 5. Conclusions

This study demonstrates that food selectivity is a highly prevalent and transdiagnostic feature in children and adolescents referred to a tertiary pediatric nutrition center, affecting the majority of the cohort regardless of the primary referral diagnosis or BMI status. A substantial proportion of patients presented with unrecognized selective eating—food group avoidance that was clinically present but not the primary reason for referral—and shared the same behavioral correlates as children with a formal selective eating/ARFID diagnosis.

These findings indicate that BMI-based screening is insufficient to identify children at nutritional risk due to food selectivity, and that a brief, structured assessment of food group avoidance should be integrated into the routine nutritional evaluation of all children referred for pediatric nutrition care, irrespective of the primary diagnosis. The gradient in ferritin deficiency observed across groups further underscores the nutritional relevance of food selectivity beyond the formal ARFID diagnostic category.

Future prospective studies using standardized dietary assessment and systematic laboratory protocols are needed to confirm the prevalence and clinical impact of nutritional deficiencies in children with unrecognized selective eating, and to evaluate the effectiveness of transdiagnostic interventions targeting mealtime behavioral correlates in this population.

## Figures and Tables

**Table 1:** Distribution of referral diagnoses and prevalence of food selectivity across diagnostic groups (N = 417).

Referral Diagnosis	N	% of Cohort	n with Selectivity	% with Selectivity
Selective eating/ARFID	141	33.7%	129	**91.5%**
Growth faltering	88	21.1%	45	51.1%
Eating disorders	66	15.8%	33	50.0%
Obesity	49	11.8%	35	**71.4%**
Overweight	40	9.6%	28	**70** **.0%**
Other	33	7.9%	23	67.6%
**Total**	**417**	**100%**	**293**	**70.3%**

Food selectivity defined as documented avoidance of ≥1 of four food groups (vegetables, fruit, fish, legumes). ARFID = avoidant/restrictive food intake disorder. Bold values indicate ≥70% prevalence.

**Table 2:** Baseline characteristics of the three study groups (N = 417).

Characteristic	Group A (n = 141)	Group B (n = 163)	Group C (n = 113)	*p*-Value
Sex, male—n (%)	80 (56.7)	73 (44.8)	46 (40.4)	0.022
Age, years—mean ± SD	7.1 ± 3.9	9.6 ± 4.7	9.6 ± 6.0	<0.001
BMI z-score—mean ± SD	−0.73 ± 1.34	0.35 ± 1.86	−0.03 ± 1.90	<0.001
BMI category (underweight/normal/overweight/obese), n (%)	26 (20.6%)/90 (71.4%)/7 (5.6%)/3 (2.4%)	23 (15.9%)/53 (36.6%)/16 (11.0%)/53 (36.6%)	19 (22.1%)/36 (41.9%)/8 (9.3%)/23 (26.7%)	<0.001

Data are presented as n (%) for categorical variables and mean ± SD for continuous variables. *p*-values from the chi-square test (categorical) or the Kruskal–Wallis test (continuous). BMI-category counts are reported in the order underweight/normal weight/overweight/obese. Age, sex and BMI distributions were recomputed from the full anonymized dataset.

**Table 3:** Prevalence of food selectivity by category and group (N = 417).

	Group A n/N (%)	Group B n/N (%)	Group C n/N (%)	*p* (A vs. B)	*p* (B vs. C)
Vegetables	119/141 (84.4%)	127/163 (77.9%)	— *	0.198	**<0.001**
Fruit	98/141 (69.5%)	83/163 (50.9%)	— *	**0.001**	**<0.001**
Fish	109/141 (77.3%)	97/163 (59.5%)	— *	**0.001**	**<0.001**
Legumes	105/141 (74.5%)	89/163 (54.6%)	— *	**<0.001**	**<0.001**
**≥1 food group (any selectivity)**	**129/141 (91.5%)**	**163/163 (100%)**	**0/113 (0%)**	— †	— †
**All 4 food groups**	**78/141 (55.3%)**	**43/163 (26.4%)**	**0/113 (0%)**	**<0.001**	**<0.001**

* Group C is defined by the absence of selectivity for all four groups; individual category rates are 0% by definition. † By definition, Group B comprises only children with selectivity for ≥1 food group (100%) and Group C only those with no selectivity (0%); between-group comparisons for the “≥1 food group (any selectivity)” row are therefore tautological and not reported. Chi-square test for comparisons between groups. Bold *p*-values indicate statistical significance (*p* < 0.05).

**Table 4:** Nutritional deficiencies by group.

Biomarker	Group A n/N (%)	Group B n/N (%)	Group C n/N (%)	*p*
Ferritin deficiency	28/87 (32.2%)	22/123 (17.9%)	9/83 (10.8%)	**0.002**
Vitamin D deficiency	27/80 (33.8%)	42/115 (36.5%)	21/78 (26.9%)	0.374
Vitamin C deficiency	21/66 (31.8%)	18/74 (24.3%)	12/56 (21.4%)	0.391
Folate deficiency †	26/35 (74.3%)	22/29 (75.9%)	13/22 (59.1%)	0.363

Deficiency recorded as binary variable (yes/no) per age-appropriate laboratory reference ranges. High proportion of missing data reflects non-uniform laboratory testing across clinical settings and referral types. Chi-square test. † Folate data available in only 35 (A), 29 (B), and 22 (C) patients. Bold *p*-values indicate statistical significance (*p* < 0.05).

**Table 5:** Behavioral and lifestyle variables by group.

Variable	Group A n/N (%)	Group B n/N (%)	Group C n/N (%)	*p*
*Screen and digital habits*				
Screen use during meals	59/101 (58.4%)	68/119 (57.1%)	25/74 (33.8%)	**0.002**
Screen time > 2 h/day	43/103 (41.7%)	66/124 (53.2%)	33/75 (44.0%)	0.188
*Dietary habits*				
Ultra-processed food consumption	81/130 (62.3%)	98/154 (63.6%)	41/99 (41.4%)	**<0.001**
Sugar-sweetened beverages	37/120 (30.8%)	37/145 (25.5%)	15/97 (15.5%)	**0.031**
≥1 family meal/day	123/125 (98.4%)	151/156 (96.8%)	102/107 (95.3%)	0.401
5 structured meals/day	87/139 (62.6%)	103/163 (63.2%)	80/112 (71.4%)	0.269
Breakfast consumption	120/139 (86.3%)	128/163 (78.5%)	95/111 (85.6%)	0.14
Meal preparation with child	29/105 (27.6%)	18/122 (14.8%)	20/78 (25.6%)	**0.043**
*Sleep and physical activity*				
Co-sleeping	58/96 (60.4%)	54/114 (47.4%)	21/67 (31.3%)	**<0.001**
Irregular sleep–wake rhythm	20/108 (18.5%)	33/127 (26.0%)	11/77 (14.3%)	0.109
Any physical activity (daily activity or organized sport)	54/82 (65.9%)	84/118 (71.2%)	56/75 (74.7%)	0.471

Chi-square test for all comparisons. n/N = number with the characteristic/number with available data. Bold *p*-values: *p* < 0.05.

**Table 6:** Adjusted associations between behavioral correlates and food selectivity.

Behavioral Variable	Adjusted OR (95% CI)	*p*-Value	Cramér’s V (95% CI)
Screen use during meals	2.50 (1.47–4.26)	0.001	0.21 (0.11–0.32)
Screen time > 2 h/day	1.57 (0.87–2.84)	0.137	0.11 (0.03–0.23)
Ultra-processed food consumption	2.87 (1.79–4.58)	<0.001	0.19 (0.10–0.29)
Sugar-sweetened beverages	2.32 (1.25–4.29)	0.007	0.14 (0.06–0.24)
5 structured meals/day	0.61 (0.38–0.98)	0.040	0.09 (0.03–0.18)
Meal preparation with child	0.60 (0.33–1.12)	0.107	0.15 (0.07–0.28)
Co-sleeping	2.02 (1.14–3.58)	0.016	0.22 (0.12–0.34)
Any physical activity (composite)	0.68 (0.37–1.27)	0.227	0.07 (0.02–0.20)

Adjusted odds ratios were estimated by multivariable logistic regression with food selectivity (avoidance of ≥1 food group) as the outcome, adjusted for age and sex. Cramér’s V refers to the three-group comparison (Groups A, B and C), with 95% bootstrap confidence intervals. Organized sport and daily physical activity were combined into a single composite measure of any physical activity.

## Data Availability

Data supporting the findings of this study are available from the corresponding author upon reasonable request.
